# Analysis of Unused Organ Donors in the Netherlands: Older Donor Age Associated With Higher Risk of Non-Utilization

**DOI:** 10.3389/ti.2025.14157

**Published:** 2025-03-11

**Authors:** K. A. Chotkan, M. A. Kuiper, I. P. J. Alwayn, M. B. A. Heemskerk, A. E. Braat, N. E. Jansen

**Affiliations:** ^1^ Department of Surgery, Division of Transplantation, Leiden University Medical Center, Leiden, Netherlands; ^2^ Dutch Transplant Foundation, Leiden, Netherlands; ^3^ Department of Intensive Care, Medical Center Leeuwarden, Leeuwarden, Netherlands; ^4^ Transplant Center, Leiden University Medical Center, Leiden, Netherlands

**Keywords:** organ donation, utilization, marginal donor, organ transplantation, end of life care

## Abstract

This study aims to provide objective evidence for the subjectively observed increase in non-utilized donors and to investigate whether they share common risk factors, hypothesizing that the aging of the donor population may be a possible explanation. All referred deceased donors in the Netherlands between 2018 and 2023 were analyzed. A utilized donor was defined as a referred donor that resulted in at least one transplanted organ. A non-utilized donor was defined as a donor from whom no organ was transplanted as a result of the cessation. In total, 2,235 donors were defined as referred; 1,618 donors were utilized and 617 were non-utilized. A significant increase in referred donors aged >66 years was observed, together with an increase of 51% in non-utilized donors. The most frequent reasons for not utilizing a donor were found to be an agonal phase > 2 hours in DCD donors (45%) and an unacceptable medical history at screening (22%). Multivariable logistic regression analysis showed that increasing donor age (age 66–75 years OR 1.81, 95% CI 1.09–3.00), DCD donors (OR 4.37 95% CI 3.24–5.89, p < 0.01), history of hypertension (OR 1.29 95% CI 1.01–1.66, p = 0.04) and/or diabetes (OR 2.48 95% CI 1.75–3.51, p < 0.01) were associated with non-utilization. Non-utilized donors are significantly older, are more often DCD donors and have more co-morbidities, confirming the hypothesis that these donors are the more marginal donors.

## Graphical Abstract

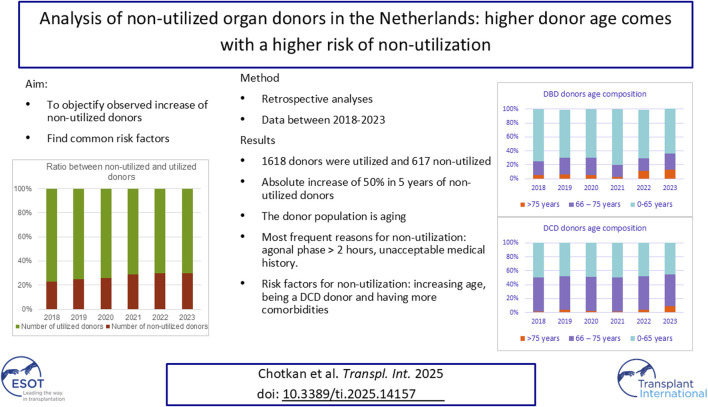

## Introduction

Donor organs are scarce, leading to an imbalance between their availability and the number of patients on the waiting list. Therefore, making optimal use of the potential donor population is important. On the other hand, donor acceptance criteria and extensive donor screening are necessary to protect vulnerable transplant recipients from disease transmission and to select organs of acceptable quality for transplantation [1]. In the Netherlands, organ donation is possible after the determination of death by neurological criteria (Donation after Brain Death, DBD) or by circulatory criteria (donation after circulatory determination of death, DCDD or DCD). DCD is only possible after withdrawal of life-sustaining treatment (WLST), the so-called controlled DCD [2, 3]. Identifying potential donors in the Netherlands is mostly performed by intensivists. After identifying a potential donor they contact “organ donation coordinators,” designated professionals who organize the organ donation procedure. In the Netherlands organs are first allocated (placed) and accepted for a specific recipient before they are procured.

In the last few years the number of referred donors has been increasing, leading to the highest number of deceased organ transplantations in the Netherlands in 2023 [4–6]. Many efforts are being made by transplant physicians to maximize the use of donor organs from the potential donor population, such as the advances in organ preservation by machine perfusion following kidney, liver, heart and lung donation [7–10]. Since 2016, all kidneys transplanted in the Netherlands have been routinely placed on hypothermic machine perfusion [9, 11, 12]. Since 2017, extended criteria for lungs and since 2022 certain DCD livers transplanted in the Netherlands are placed on machine perfusion in the transplant center, in accordance with national regulations [8, 13, 14]. In 2021, DCD heart donation was introduced in the Netherlands, with all DCD hearts placed on machine perfusion [15].

Together with this innovation the criteria for donor acceptance have been expanded. In 2018, the age limit of 60 years for DCD-liver donation, was abolished. There has also been no age limit for kidney and liver donation for DBD donors for several years. In 2019 the age limit for heart donation in the Netherlands was raised from 65 to 70 years and in July 2020 the age limit of 75 years for lung donation was also abolished [16]. Expanding the donor criteria may lead to the acceptance of older and more marginal donors [5, 17]. These patients are at a higher risk of comorbidities, diminished organ quality and possible medical contraindications, which could lead to an increased likelihood of discontinuation of the procedure [18, 19].

Parallel to these changes the government has been trying to increase the number of organ transplantations. Therefore on 1 July 2020, a law was implemented in the Netherlands that effectively changed the consent system from “Opt-in” to “Opt-out.” In an “Opt-in” consent system, a donation can only take place when there is explicit consent, from the donor (Donor Registry) or the relatives. In an “Opt-out” system, consent for donation is presumed unless there is an objection to donation registered in the Donor Registry [20].

Currently, Intensive Care Units (ICUs) and procurement teams in the Netherlands have observed a substantial increase in procedures that do not result in the transplantation of organs. This study aims to determine whether there is a change in the percentage of donors from whom at least one organ is being transplanted and to investigate whether non-utilized donors share common risk factors. The hypothesis is that unused organs belong to referred donors who tend to be older and more marginalized.

## Patients and Methods

### Study Design and Population

This study is a retrospective analysis of all deceased organ donors in the Netherlands who were referred to the Organ Donor Coordinator between 1 January 2018 and 31 December 2023. The process between donor recognition by the intensive care physician and referral to the Organ Donor Coordinator (and finally to Eurotransplant) is described in Supplementary Figure S1. Donor referral criteria are described in Supplementary Table S1. An actual donor was defined as a referred donor in whom an operative incision was made with the intent of organ recovery for the purpose of transplantation or from whom at least one organ was recovered for the purpose of transplantation. A utilized donor was defined as a referred donor that resulted in at least one transplanted organ [21]. A non-utilized donor was defined as a donor from whom no organ was transplanted, either due to the cessation of the procedure during screening, allocation, procurement or rejection at the transplant center.

A utilization rate for each donor organ type was also calculated annually by dividing the number of transplanted organs by the number of organs referred.

Data on Dutch organ donation procedures were retrieved from the Eurotransplant Data system (Donor Data) and the Organ Procurement Information (OPI) Database of the Dutch Transplantation Foundation. Donor reports were reviewed to analyze whether a procurement procedure occurred and whether at least one organ was transplanted. The number of donors referred, the number of utilized donors, and the number of non-utilized donors were compared within the selected period.

This study was conducted in accordance with the World Medical Association-Declaration of Helsinki and the Declaration of Istanbul.

### Statistical Analyses

The characteristics of all referred donors were analyzed across different years and compared between utilized and non-utilized donors. Continuous data were displayed as mean ± standard deviation (SD). Categorical data were presented as absolute numbers and percentages (%). The Kolmogorov-Smirnov test was used to determine whether continuous variables had a normal distribution. Continuous variables with a normal distribution were analyzed using parametric tests, otherwise non-parametric tests were used. Differences between categorical data were assessed using Chi-square tests. To assess risk factors for not utilizing a donor, binary logistic regression analysis was performed. Initially, each variable was analyzed using a univariable logistic regression model, followed by a multivariate model. A p-value less than 0.05 was considered statistically significant. For statistical analyses, IBM SPSS Statistics for Windows was used (IBM Corp. Released 2022. Version 29.0).

## Results

### Changes in the Donor Pool

**FIGURE 1:**
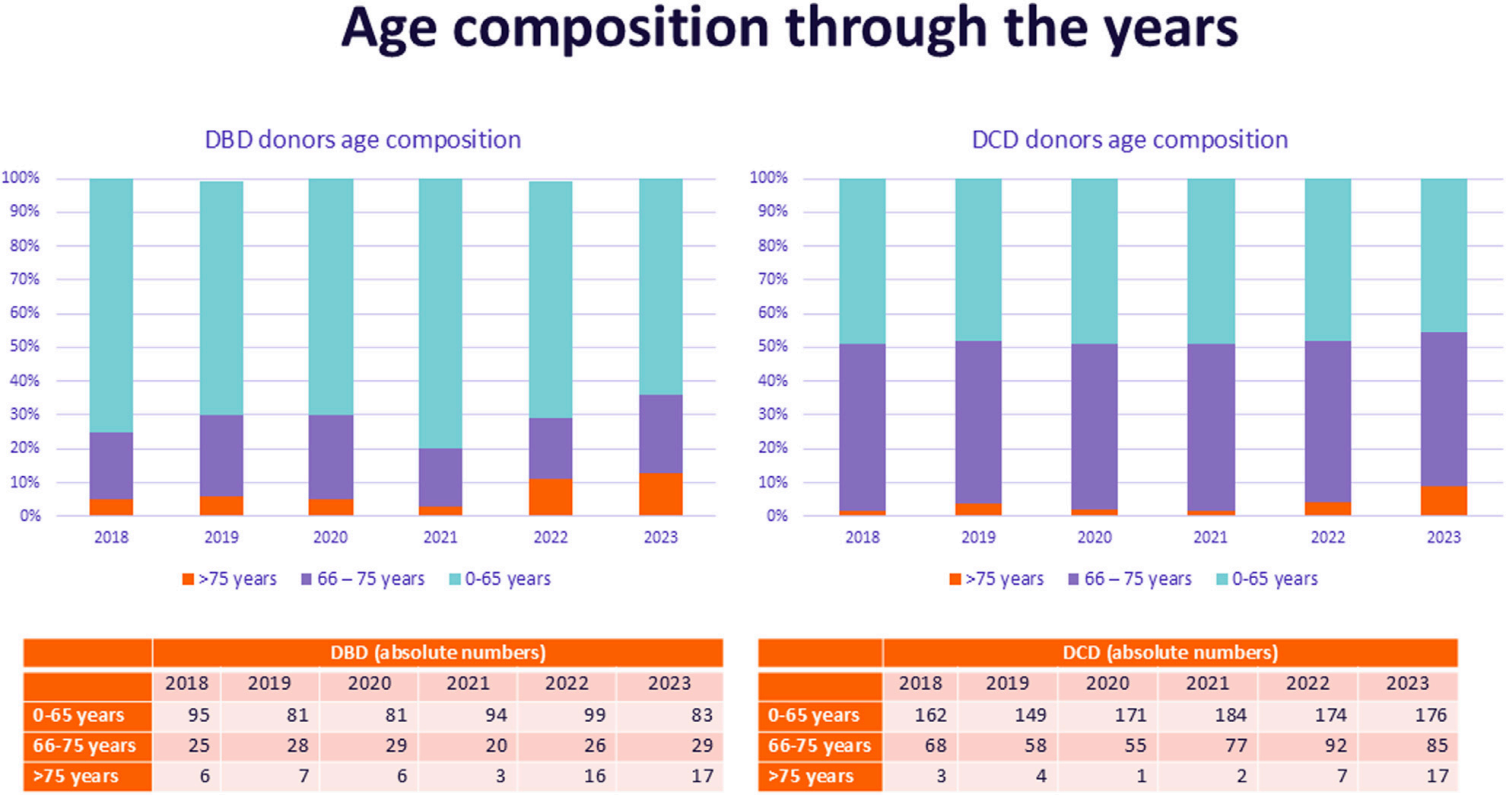
Age composition of referred donors over the years, stratified by donor type.

Between 1 January 2018, and 31 December 2023, a total of 2235 potential donors were referred. Comparing donor characteristics between 2018 and 2023 the mean donor age increased from 54 years to 57 years (although not statistically significant). When categorized by age, a significant increase was observed in the number of donors aged between 66–75 years and >75 years in 2022 and 2023 (Table 1; Figure 1). Furthermore there was an increase observed in DCD donors from 65% to 69%. There was a significant increase in the number of donors with a history of hypertension and donors with a history of malignancy.

**TABLE 1:** Donor characteristics of all referred donors, stratified by year.

	2018	2019	2020	2021	2022	2023	P-value
Number of referred donors	359	327	343	382	414	410	NA
Number of actual donors^a^	n = 283 (79%)	n = 254 (78%)	n = 256 (75%)	n = 276 (72%)	n = 298 (72%)	n = 302 (74%)	NA
Number of utilized donors^a^	n = 276 (77%)	n = 244 (75%)	n = 252 (74%)	n = 272 (71%)	n = 289 (70%)	n = 285 (70%)	NA
Number of non-utilized donors^a^	83 (23%)	83 (25%)	91 (26%)	110 (29%)	125 (30%)	125 (30%)	NA
Donor type							p = 0.65
DBD	35%	36%	34%	31%	34%	31%	
DCD	65%	64%	66%	69%	66%	69%	
Age, mean	54 ± 17	54 ± 17	55 ± 15	54 ± 16	56 ± 16	57 ± 17	p = 0.06
0–15 years	2%	2%	2%	2%	2%	1%	**p < 0.01**
16–25 years	8%	5%	6%	6%	6%	5%	
26–35 years	4%	9%	6%	7%	4%	7%	
36–45 years	9%	8%	9%	8%	7%	7%	
46–55 years	20%	19%	22%	18%	20%	16%	
56–65 years	28%	27%	30%	32%	27%	27%	
66–75 years	26%	26%	25%	26%	28%	28%	
>75 years	3%	3%	2%	1%	6%	8%	
Ender, male	59%	55%	57%	57%	55%	55%	p = 0.31
BMI	26 ± 5	26 ± 5	26 ± 5	26 ± 5	26 ± 5	27 ± 12	**p = 0.05**
<18.5	4%	2%	2.5%	3%	4.5%	2%	p = 0.88
18.5–25	43%	43%	42%	41%	44%	43%	
25–30	40%	39%	38%	39%	34%	36%	
30–35	10%	11%	13%	14%	12%	14%	
35–40	3%	4%	5%	3%	5%	4%	
Cause of death							p = 0.60
CVA	46%	46%	47%	48%	48%	48%	
Cardiac event	18%	22%	18%	21%	23%	22%	
Trauma	21%	16%	20%	17%	16%	14%	
Other	15%	16%	15%	14%	13%	17%	
History of hypertension	30%	28%	26%	27%	28%	32%	**p = 0.04**
History of diabetes	8%	9%	9%	10%	10%	9%	p = 0.84
History of smoking	56%	53%	54%	52%	55%	60%	p = 0.22
History of malignancy	5%	4%	3%	5%	6%	8%	**p = 0.03**
ASAT	111 ± 332	95 ± 188	92 ± 199	82 ± 100	84 ± 154	73 ± 107	p = 0.17
ALAT	95 ± 368	92 ± 244	77 ± 137	74 ± 109	64 ± 87	61 ± 79	p = 0.12
Total bilirubin	11 ± 8	11 ± 9	11 ± 9	11 ± 8	11 ± 20	9 ± 9	p = 0.30
CMV, IgG positive	44%	47%	40%	47%	45%	47%	p = 0.56
EBV, IgG positive	73%	71%	72%	68%	67%	71%	p = 0.73

Values are presented as mean, ±standard deviation, or as percentage. Significant differences are in bold. An Anova test was used to analyze the differences in mean age and BMI between the groups. In all other cases, a chi-square test was used.

^a^ As the percentage of the total number of referred donors.

**FIGURE 2:**
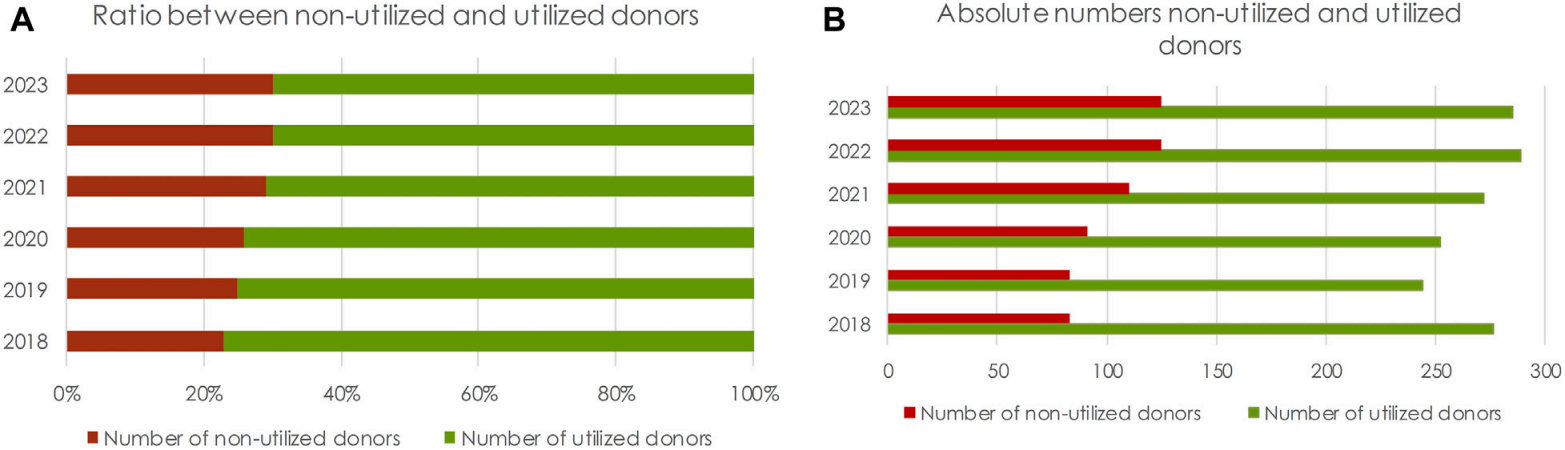
**(A)** Ratio of non-utilized to utilized donors, stratified by year. **(B)** Absolute number of non-utilized donors and utilized donors.

Of the 2235 potential donors referred, 1,670 (75%) were actual donors (donation procedures that led to a procurement procedure) and 1,618 (72.4%) resulted in the transplantation of at least one organ; 617 donors were not utilized. During this period the number of referred donors increased by 14% from 359 in 2018 to 410 in 2023 (Table 1). However, the number of utilized donors increased by only 3% from 276 procedures in 2018 to 285 procedures in 2023. The absolute number of non-utilized donors increased by 51% (83 in 2018 and 125 in 2023). The percentage of utilized donors decreased from 77% in 2018 to 70% in 2023 (Figures 2A, B; Table 1).

No significant differences were observed in laboratory values (creatinine, ASAT, ALAT, total bilirubin) or virology results (IgG CMV, IgG EBV).

### DBD Versus DCD Donors

When comparing donor characteristics between DBD and DCD donors, the age groups of donors 56–65 years, 66–75 years and >75 years were significantly higher in DCD donors (Table 2; Figure 1). The cause of death was more often Cerebral Vascular Accident (CVA) in DBD donors, while cardiac-related death was more frequently the cause of death in DCD donors (Table 2).

**TABLE 2:** Donor characteristics, comparing DBD and DCD donors.

	DBD donors	DCD donors	P-Value	Missing data
Number of donors	n = 745	n = 1,490		
Donor age	54 ± 17	56 ± 16	**p = 0.02**	0%
0–15 years	7%	2%		
16–25 years	2%	6%		
26–35 years	7%	6%		
36–45 years	9%	8%		
46–55 years	20%	18%		
56–65 years	26%	30%		
66–75 years	21%	29%		
>75 years	7%	2%		
Donor gender, male	48%%	60%	**p < 0.01**	0%
Donor cause of death			**p < 0.01**	0%
CVA	67%	37%		
Cardiac event	8%	27%		
Trauma	15%	18%		
Other	9%	18%		
BMI			p = 0.10	7.5%
<18.5	3%	3%		
18.5–25	47%	41%		
25–30	35%	39%		
30–35	11%	13%		
35–40	4%	4%		
Donor history of hypertension	32%	27%	**p = 0.04**	5%
Donor history of diabetes	8%	9%	p = 0.19	2%
Donor history of smoking	53%	55%	p = 0.47	6%
Donor history of malignancy	6%	5%	p = 0.47	6%
Blood group			p = 0.34	0%
O	45%	46%		
A	40%	42%		
B	12%	9%		
AB	4%	4%		

An Anova test was used to analyse the differences in mean age and BMI between the groups. In all other cases, the chi-squared test was used.

Significant differences in bold.

In DBD donors the percentage of non-utilized donors was found to be 13%, compared to 35% in DCD donors (Table 3). Table 3 shows the ratio of utilized to non-utilized donors by donor characteristic (stratified by donor type). In DBD donors, the percentage of non-utilized donors was significantly higher in donors aged >56 years. This was also seen in DCD donors; in DCD donors >75 years, 50% of the donors were not utilized (Table 3).

**TABLE 3:** Ratio of utilized to non-utilized donors per donor characteristic. The p-value indicates whether there is a significant difference in the ratio of utilized/non-utilized within a certain group.

	DBD			DCD		
	Utilized/non-utilized		p-value^a^	Utilized/non-utilized		p-value
Number of donors	N = 645	N = 100		N = 973	N = 517	
	87%	13%		65%	35%	
Donor age			**p = 0.02**			**p < 0.01**
0–15 years	88%	12%		71%	29%	
16–25 years	94%	6%		83%	17%	
26–35 years	93%	7%		71%	29%	
36–45 years	90%	10%		71%	29%	
46–55 years	92%	8%		68%	32%	
56–65 years	84%	16%		67%	33%	
66–75 years	82%	18%		57%	43%	
>75 years	76%	24%		50%	50%	
Donor gender			p = 0.05			p = 0.97
Male	84%	16%		65%	35%	
Female	89%	11%		66%	34%	
Donor cause of death			p = 0.62			**p < 0.01**
CVA	86%	14%		63%	17%	
Cardiac event	87%	13%		61%	39%	
Trauma	90%	10%		72%	28%	
Other	86%	14%		71%	29%	
BMI			p = 0.78			p = 0.08
<18.5	92%	8%		82%	18%	
18.5–25	91%	9%		70%	30%	
25–30	89%	11%		68%	32%	
30–35	85%	15%		64%	36%	
35–40	90%	10%		78%	22%	
Donor history of hypertension			**p = 0.03**			**p < 0.01**
Yes	89%	11%		58%	42%	
No	93%	7%		70%	30%	
Donor history of diabetes			**p < 0.01**			**p < 0.01**
Yes	65%	35%		47%	53%	
No	89%	11%		53%	47%	
Donor history of smoking			**p < 0.01**			**p < 0.01**
Yes	91%	9%		66%	34%	
No	91%	9%		74%	26%	
Donor history of malignancy			**p < 0.01**			**p = 0.01**
Yes	82%	18%		69%	31%	
No	92%	8%		69%	31%	
Blood group			p = 0.29			p = 0.52
O	91%	9%		67%	33%	
A	89%	11%		66%	34%	
B	84%	16%		61%	39%	
AB	88%	12%		63%	37%	

^a^ A chi-square test was used to investigate the differences between the groups.

Significant differences in bold.

When further stratifying for specific reasons for not utilizing a donor, especially in younger DCD donors aged 0–15 years, consent was found to be more often withdrawn (the percentage of non-utilized donors was found to be 17% in this group) (Table 4). In particular, donors >66 years were more frequently non-utilized because their medical history was not acceptable. In total, 21% of DCD donors aged 66–75 years were not utilized because of an agonal phase >2 h.

**TABLE 4:** Age distribution of donors between utilized donors and certain categories of non-utilized donors. (Every row totals 100%).

DBD	Utilized donors	Medical history/unstable donors	Medical virology	Permission withdrawn/no recipients	Cancellation due to findings during procurement
Number of donors	644	50	12	19	17
Donor age					
0–15 years	88%	0%	6%	6%	0%
16–25 years	93%	6%	0%	1%	0%
26–35 years	94%	2%	2%	2%	0%
36–45 years	89%	6%	2%	3%	0%
46–55 years	92%	3%	1%	1%	3%
56–65 years	84%	7%	3%	3%	3%
66–75 years	83%	10%	1%	3%	4%
>75 years	75%	15%	0%	8%	2%

DCD	Utilized donors	Medical history/unstable donors	Medical virology	Permission withdrawn/no recipients	Cancellation due to findings during procurement	Agonal phase > 2 h
Number of donors	972	144	19	64	35	253
Donor age						
0–15 years	71%	4%	0%	17%	4%	4%
16–25 years	79%	5%	2%	5%	1%	9%
26–35 years	70%	6%	0%	8%	1%	15%
36–45 years	73%	9%	1%	5%	1%	11%
46–55 years	67%	7%	1%	6%	1%	18%
56–65 years	68%	11%	1%	2%	1%	16%
66–75 years	56%	13%	2%	4%	5%	21%
>75 years	54%	11%	4%	4%	11%	18%

### Referred Donors, Not Resulting in a Procurement Procedure

**FIGURE 3:**
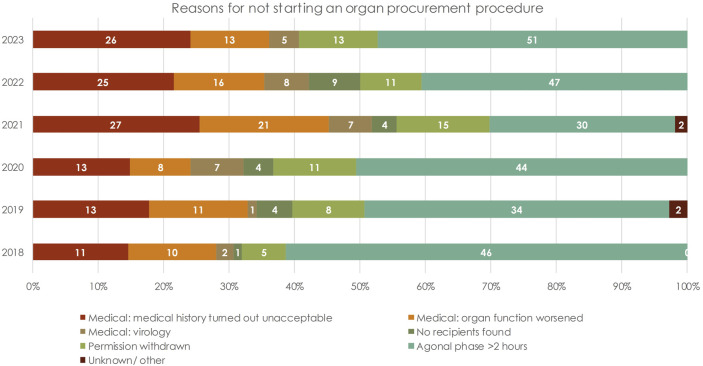
Reasons for not starting an organ procurement procedure, stratified by year. Each bar shows the absolute number of cases.

In total, 565 referred donors did not become donors. The most frequent reason for this was “agonal phase >2 h in DCD donors” (n = 253/565, 45%), followed by “medical history found to be unacceptable during screening” (n = 125/565, 22%) and “worsening of the organ function during the screening or allocation process” (n = 68/565, 12%) (Figure 3). In total, 5% of the referred potential donors (31/565) were not utilized because of “medical virology,” of which 54% (n = 17/31) was related to SARS-CoV-2. For 23 donors (4%) “no suitable recipient” was found.

### Unacceptable Medical History

In total, 22% (125/565) of the referred donors who did not become actual donors were rejected because their medical history was deemed unacceptable for transplantation. Detailed analysis showed that this was frequently due to the quality of the donor (50/125, 40%), which was insufficient to continue the organ donation process, i.e., because of comorbidity (diabetes, hypertension, BMI) in combination with poor organ function. In 12% (n = 15) of the cases the procedure was canceled because of a proven malignancy in the patient’s medical history, and in 30% (n = 38) because of suspected malignancy during the screening process (in 28 cases, 22%, this was specifically reported as seen on imaging). In 14% of the cases, there was another contraindication (mass transfusion, risk behavior, deviation on imaging abnormality not further specified, improvement in EMV score). In 3% of the subjects, the medical contraindication for organ donation was not further specified in the database.

### Procurement Started; No Organ Transplanted

In total, 52 procurement procedures were started, of which no organs were transplanted (Table 5). In 65% of the cases (n = 34/52), this was because of organ quality. Donors, whose organs were rejected during or after procurement because of the quality, were accepted for only one type of organ in 44% (n = 23) of the cases (liver n = 15, kidney n = 6, lung n = 1, heart n = 1). In 2023 an increase was seen in the number of donation procedures canceled during the procurement because of organ quality; 12 cases, of which seven were exclusively liver-only donors.

**TABLE 5:** Number of procedures terminated during procurement, stratified by reason and per year.

	Malignancy found during procurement	Poor organ quality seen during or after procurement	Other medical contraindication
2018	0	8	0
2019	2	7	1
2020	3	0	1
2021	2	2	0
2022	4	5	0
2023	2	12	3
Total	13	34	5

Despite screening, malignancy was found in 13 donors (25%). Other reasons for cessation of the donation procedure were bowel perforation seen during procurement (n = 2), bowel ischemia seen during procurement (n = 1), or cannulation of the donor being impossible (n = 1) and rejection of the organ by the transplant center after procurement (n = 1).

### Utilization Rate by Organ Type

In Supplementary Figure S2 the utilization rate per organ type is represented. The utilization rate was found to be highest for kidneys and the lowest for pancreas grafts, with both remaining relatively constant over time. For liver grafts, we observe a decrease in utilization rate from 2021. A decrease in the utilization rate of heart donation was observed in 2021.

#### Risk Factors for Non-Utilization

In univariable logistic regression analysis, donors aged between 66 and 75 years had a 3.98 times higher risk of not being utilized compared to donors aged 16–25 years (OR 3.98, CI 95% 2.33–6.80, p < 0.01) (Table 6). Also, DCD donors had a 3.43 higher risk of not being utilized compared to DBD donors (OR 3.43, CI 95% 2.71–4.34, p = <0.01) (Table 6). Additionally, in univariable logistic regression, donor male gender, increasing age, death by cardiac event, BMI > 25, and donor history of hypertension and diabetes were shown to be significant risk factors for not utilizing a donor. In multivariable logistic regression analysis of DCD donors, increasing donor age, and history of hypertension and diabetes were found to be significantly associated with a higher risk of non-utilization (Table 6).

$$\begin{array}{c} \text{Using the logistic regression formula P (chance of donor non-utilization)}=\\ \frac{e^{-2.763+1.474*donortype+\beta *age+\beta \;BMI+0.258*hypertension+0.907*diabetes+\beta *cause\;of\;death}}{e^{-2.763+1.474*donortype+\beta *age+\beta \;BMI+0.258*hypertension+0.907*diabetes+\beta *cause\;of\;death}+1\;} \end{array}$$

**TABLE 6:** Odds ratios of risk factors for risk of not utilizing a donor.

	OR, univariable model		OR, multivariable model	Β- coefficient	
Donor type		**p < 0.01**			**p < 0.01**
DBD	1.00		1.00		
DCD	3.43 [2.71–4.34]		4.37 [3.24–5.89]	1.474	
Donor age		**p < 0.01**			**p < 0.01**
0–15 years	1.99 [0.81–4.92]		1.41.03 [0.43–4.39]	0.344	
16–25 years	1.00		1.00		
26–35 years	1.75 [0.91–3.38]		1.22 [0.66–2.26]	0.201	
36–45 years	1.93 [1.04–3.59]		0.99 [0.55–1.81]	0.006	
46–55 years	2.12 [1.21–3.69]		1.16 [0.69–1.96]	0.148	
56–65 years	2.62 [1.53–4.48]		1.30 [0.78–2.16]	0.264	
66–75 years	3.98 [2.33–6.80]		1.81 [1.09–3.00]	0.593	
>75 years	3.50 [1.78–6.85]		2.98 [1.45–6.17]	1.094	
Donor gender		**p < 0.01**			p = 0.19
Female	1.00		1.00		
Male	1.24 [1.02–1.49]		1.08 [0.87–1.36]	0.082	
BMI		**p = 0.049**			p = 0.19
<18.5	0.59 [0.28–1.20]		0.58 [0.26–1.30]	−0.541	
18.5–25	1.00		1.00	0.021	
25–30	1.20 [0.95–1.50]		1.02 [0.80–1.31]	0.152	
30–35	1.43 [1.04–1.95]		1.16 [0.83–1.63]	0.152	
35–40	0.91 [0.52–1.58]		0.60 [0.33–1.09]	−0.516	
History of hypertension	1.58 [1.29–1.93]	**p < 0.01**	1.29 [1.01–1.66]	0.258	**p = 0.04**
History of diabetes	2.78 [2.07–3.75]	**p < 0.01**	2.48 [1.75–3.51]	0.907	**p < 0.01**
Cause of death		**p < 0.01**			
CVA	1.00		1.00		p = 0.14
Cardiac event	1.57 [1.24–1.99]		1.08 [0.82–1.43]	0.080	
Trauma	0.82 [0.62–1.08]		0.78 [0.56–1.08]	−0.254	
Other	1.01 [0.76–1.31]		0.77 [0.55–1.08]	−0.260	
Blood group					
O	1.00	p = 0.39			
A	1.12 [0.90–1.38]				
B	1.32 [0.94–1.84]				
AB	1.20 [0.71–2.02]				

^a^ In the multivariable analysis donor- gender, -age, -type, history of hypertension, history of diabetes, and cause of death are all added at once in the same model.

Signifcant differences in bold.

For each donor the chance of not being utilized was calculated. The “β” coefficient can be found in Table 6. For example, a female DBD donor aged 30 years, with a BMI of 26, no hypertension and diabetes, and a cause of death of trauma has a 6% chance of not being utilized. A male DCD donor aged 76 years with a BMI of 31, a history of hypertension and diabetes and a cause of death of cardiac event has a 78% chance of not being utilized.

The binary logistic regression analysis was repeated, excluding those DCD donors with an agonal phase exceeding 2 h. The results of this analysis are presented in Supplementary Table S2. Comparing Supplementary Table S2 with Table 6, the OR for being a DCD donor decreased from 4.37 to 1.78. Also in this multivariable model, male gender is associated with a significantly higher risk of non-utilization, whereas a history of hypertension is not (which is the opposite in the model including DCD donors with an agonal phase exceeding 2 h).

## Discussion

This study aimed to investigate whether non-utilized donors share common risk factors. The present data show that although the absolute number of organ donation procedures and transplantations has increased in the Netherlands between 2018 and 2023, the percentage of non-utilized donors also increased from 23% to 30%. Non-utilized donors are significantly older, are more often DCD donors and have more co-morbidities, confirming the hypothesis that these donors are more marginalized. In other words, risk factors for not utilizing a donor are DCD donation, donors with increasing age, hypertension and diabetes as comorbidity, as shown by logistic regression analyses (Table 6). Donors older than 66 years and older than 75 years have a risk of non-utilization, with odds ratios of 3.98 and 3.50, respectively (Table 6). Furthermore, DCD donors have a 3.43 times higher risk of non-utilization compared to DBD donors, caused in part by the guidelines regarding the length of the agonal phase in DCD donation [22].

Using the formula described above, the risk of non-utilization can be calculated for every donor (representing the chance of non-utilization), raising the question at what percentage the line should be drawn. A male DCD donor aged 76 years with a BMI of 31, a history of hypertension and diabetes and a cause of death of a cardiac event, has a 78% chance of not being utilized. From a transplantation perspective, this model would suggest that in 22% of the cases this does lead to an organ transplantation, increasing the absolute number of utilized donors. With an aging pool of potential donors this group of marginal donors will most likely increase in the future. This makes it difficult to make recommendations regarding changes in donor acceptance criteria based on the results of this study. We therefore did not validate the predictive value of this model. Also, the model is unlikely to be applicable in countries with a lower proportion of DCD donors and should therefore be used with caution. The impression that an increase in the number of referred donors does not lead to an equivalent increase in the number of donors used has also been mentioned by Neuberger et al., who investigated donor utilization rates in the UK [23]. Nevertheless, to our knowledge, this is the first study describing a model for the risk of non-utilization.

Since 2021 there has been an increase in the number of donors not utilized because their medical history has been found to be unacceptable, in addition to the group of donors not utilized because of worsening organ function. This could be a consequence of the broadening of donor acceptance criteria in previous years, especially the acceptance of older donors. Figure 1 illustrates the growing acceptance of the elderly donor. Donors aged above 75 years were increasingly referred in 2022 and 2023 compared to the previously years. This is supported by Table 4, which shows that the medical history of older donors is particularly unacceptable. Older donors have a higher risk of developing a malignancy, and consequently of having an undetected malignancy revealed during screening or procurement [24, 25]. This was also observed in our study, where in 38 of the cases cessation of the donor procedure was related to a (possible) malignancy, detected during screening or procurement. In fifteen cases the donor was rejected due to the presence of a malignancy in their medical history. Having a malignancy is not a general (absolute) contraindication, but the acceptance of an organ depends on the type of malignancy. Certain brain tumors are widely accepted [22]. Donor-transmitted cancer has a poor prognosis, and requires cancer treatment, therefore transplant centers are reluctant to accept a donor with a malignancy in their medical history [26].

Remarkably, the number of procurement procedures involving organs rejected due to poor quality was highest in 2023: seven out of the twelve donors rejected for donor quality were exclusively liver-only donors, which is reflected in the decline in liver utilization rate observed since 2021 (Supplementary Figure S2). This is probably due to the wider acceptance of liver donors, because of the introduction of normothermic perfusion, and the removal of the age limit for liver donation in DCD donors [8, 22]. Machine perfusion in liver donation provides an opportunity to test graft function, making transplant physicians more willing to accept marginal liver donors and encouraging the reporting of every possible donor. From a broader perspective, machine perfusion gives the opportunity to accept marginal organs, allowing for less stringent acceptance criteria, which could result in a higher referral rate. Nevertheless, despite the decrease in utilization rates, the widening of the acceptance criteria has led to an increase in the number of organ transplantations in the Netherlands [4–6].

In particular, the group of donors ceased during or after procurement, along with the group of donors with an agonal phase >2 h, has a major impact on all parties involved and costs (i.e., operating room and machine perfusion equipment). In addition, donors would be likely to have occupied a bed in the ICU for an extended period while all necessary diagnostics are performed. In addition to the costs, the efforts of the ICU staff and the procurement team would be wasted. Finally, the disappointment of family members would be higher if the donation procedure fails, although no qualitative research was conducted on this topic in this study. It is important to mention that this study did not focus on insights about donors with an agonal phase >2 h in whom organ donation did not take place, considering the extensive existing research on this topic, which showed no reliable predictors to ensure cardiac death <2 h [27–29]. In the Netherlands, the rate of non-utilized DCD donors due to an agonal phase of less than 2 hours is known to be higher compared to other (European) countries. Similar to the Netherlands the United Kingdom noted in a report from 2023 to 2024 that 53% of the DCD donors did not proceed to donation because of a prolonged agonal phase [30]. The discrepancy between the Netherlands and the UK versus other countries is attributed to differences in end-of-life care practices.

This study has several limitations that need to be addressed. First, the retrospective study design makes missing information inevitable, for example, not for all donors the reason for non-utilization could be retrieved. There is a certain bias affecting the number of referred donors in this study. As pointed out before, the consent system for organ donation changed from an “Opt-in” to an “Opt-out.” Before the Dutch consent system changed, less than 50% of all adults were registered in the Donor Registry, while with the Opt-out system all residents aged 18 and older are registered. As of October 2023 the registrations are as follows: 34% are registered as a “Yes, I want to be an organ donor,” and 24% with the new category “No objection” (as a consequence of no response to the call to register). [31, 32] The change in the system could be the cause of the increase in the number of referred potential donors, but it is too early to draw conclusions about the effect of the “Opt-out” consent system. Changing the consent system was accompanied by a high level of publicity and education regarding organ donation. This may have affected the number of referred potential donors in two ways; increased awareness among healthcare professionals and increased awareness among the public (encouraging people to talk to their relatives about their donation preferences).

Another factor affecting the number of referred donors is the fact that part of the study period occurred during the COVID-19 pandemic. During the first wave of the COVID-19 pandemic in March 2020, there was a decrease in the number of organ donation procedures, because the majority of the deaths in the ICU were related to SARS-CoV-2 and due to ICUs being at capacity [33]. Nonetheless, this decrease is not seen in the total number of referred donors for 2020 and 2021 (which decreased together with the change to the “Opt-out” consent system). It is important to note that in the Netherlands, during the COVID-19 pandemic, ICUs tried to maximize organ donation despite capacity constraints.

In conclusion, our study shows that the donor population, just as the general population, is aging. The increase seen in the absolute number of organ transplants is associated with an increased risk of non-utilization. In particular, older DCD donors are at risk of not being utilized. Starting a donation procedure for donors with a high risk of non-utilization requires a great deal of effort on the part of the ICU (and organ donor coordinators), and does not always lead to an organ donation and transplantation procedure. This makes donation procedures less efficient and increases the costs. To achieve high numbers of organ transplantations, donor acceptance criteria have been broadened and nearly every potential donor is referred, regardless of age, comorbidity, and medical history. This inevitably also leads to a high number of cessations of donation procedures due to the unsuitability of the donor. The acceptance of an organ for a specific recipient requires a case-by-case evaluation as a marginal donor may be suitable for a patient with high urgency on the waiting list. Thus, although every donor referral is worth the effort, the effort of the donation professionals should be balanced. Whether the increase in absolute numbers of organ transplantations is worth the price of decreased utilization rate is a calculation that each country has to make for itself.

## Data Availability

The raw data supporting the conclusions of this article will be made available by the authors, without undue reservation.

## Abbreviations

CI, Confidence Interval; DBD, Donation after Brain Death; DCD, Donation after Circulatory determination of death; ICU, Intensive Care Unit; OR, Odds Ratio; SD, Standard deviation.
