# Supplementary material for: Analysis of Unused Organ Donors in the Netherlands: Older Donor Age Associated With Higher Risk of Non-Utilization
Source: Transpl Int. 2025 Mar 11;38:14157. doi: 10.3389/ti.2025.14157 (PMC11934072; doi:10.3389/ti.2025.14157)
Supplement: Supplementary file 1 [file DataSheet1.docx]

**Figure 1:** Flowchart of the process between donor recognition by the intensive physician to referral to the Organ Donor Coordinator (and eventually Eurotransplant). Donors from step 4 (in red) and onwards are included in the current study.

**Table 1**, General and organ specific contra-indications, source: Model protocol deceased organ donation, page 17-19 [modelprotocol-versie-9-januari-2025.pdf](https://www.transplantatiestichting.nl/files/Protocollen%20en%20richtlijnen/modelprotocol-versie-9-januari-2025.pdf) [In Dutch]

| General contra-indications | | Relative contra-indications | | |
| --- | --- | --- | --- | --- |
| - Unknown identity of the patient - <24 hours adequately treated sepsis - Active tuberculosis - Active viral infection with rabies, herpes zoster or rubella - Anencephalia | | - HIV - Chronic Q-fever - Malignancies - Unknown cause of death - Melanoma | | |
| Organ specific contra-indications | |  | | |
|  | **Contra-indication** | | **Age criterium DBD** | **Age criterium DCD** |
| Kidneys | - Primary kidney disease | | No limit | ± 75 years |
| Liver | - Proven liver cirrhosis - History of hemophilia B | | No limit | **>**1 month old |
| Lungs | - Chronic obstructive lung disease (for which medication is used) - Other lung pathology - Proven aspiration - Melanoma | | No limit | No limit |
| Heart | - Angina pectoris, myocardial infarct, history of coronary bypass surgery - Severe valve disease - Malignant rhythm disorders - Cardiomyopathy - Insulin dependent diabetes mellitus | | ± 70 years | Until 58 years, weight minimum of 50 kg |
| Pancreas | - Diabetes mellitus type I - Chronic or acute pancreatitis | | Whole: ± 60 years  Islets: ± 75 years | Whole:5 until ± 50years  Islets: ± 75 years |
| Small bowel | - Bowel resection in the medical history - Inflammatory bowel disease - Celiac disease | | 1 until ± 50years |  |

**Figure 2:** Utilization rate per organ type

**Table 2:** Odds ratios of risk factors for the risk of not utilizing a donor, excluding the DCD donors with an agonal phase exceeding two hours

|  | OR, univariable model |  | OR, multivariable model |  |
| --- | --- | --- | --- | --- |
| Donor type   - DBD - DCD | 1.00  1.80 [1.40-2.31] | **p<0.01** | 1.00  1.78 [1.28-2.48] | **p<0.01** |
| Donor age  0 – 15 years  16 – 25 years  26 –35 years  36 – 45 years  46 – 55 years  56 – 65 years  66 – 75 years  >75 years | 2.12 [0.85-5.32]  1.00  1.16 [0.55-2.44]  1.46 [0.76-2.81]  1.34 [0.76-2.36]  1.85 [1.09-3.16]  2.69 [1.58-4.57]  3.22 [1.58-6.57] | **p<0.01** | 1.81 [0.50-6.49]  1.00  1.04 [0.44-2.41]  0.69 [0.30-1.56]  0.80 [0.41-1.59]  1.08 [0.57-2.04]  1.48 [0.78-2.80]  2.84 [1.23-6.54] | **p<0.01** |
| Gender   - Female - Male | 1.00  1.40 [1.11-1.78] | **p<0.01** | 1.00  1.48 [1.10-1.99] | **p<0.01** |
| BMI   - <18.5 - 18,5-25 - 25-30 - 30-35 - 35-40 | 0.58 [0.23-1.49]  1.00  0.95 [0.70-1.29]  1.35 [0.91 -2.02]  0.96 [0.47-1.92] | p=0.35 | 0.55 [0.19-1.54]  1.00  0.80 [0.41-1.59]  1.08 [0.70-1.65]  0.59 [0.28-1.24] | p=0.64 |
| History of hypertension | 1.64 [1.28-2.11]] | **p<0.01** | 1.26 [0.91=1.74] | p=0.17 |
| History of diabetes | 3.2 [2.26-4.49] | **p<0.01** | 2.81 [1.86-4.26] | **p<0.01** |
| Cause of death   - CVA - Cardiac event - Trauma - Other | 1.00  1.40 [1.03-1.82]  0.63 [0.44-0.90]  0.98 [0.70-1.38] | **p<0.01** | 1.00  1.07 [0.74-1.54]  0.59 [0.37-0.94]  0.84 [0.54-1.31] | p=0.10 |
| Blood group   - O - A - B - AB | 1.00  1.28 [0.99-1.66]  1.53 [1.04-2.27]  1.40 [0.76-2.60] | p=0.10 |  |  |
